# Dyspnea and Deception: Overcoming Diagnostic Hurdles in Pulmonary Alveolar Proteinosis

**DOI:** 10.7759/cureus.84562

**Published:** 2025-05-21

**Authors:** Ketan Jolly, Merry Mathew, Hanieh Tabatabaei, Barath Rangaswamy

**Affiliations:** 1 Department of Internal Medicine, Texas Tech University Health Sciences Center, Odessa, USA

**Keywords:** autoimmune pulmonary alveolar proteinosis, bronchoalveolar lavage, crazy paving pattern, granulocyte macrophage colony stimulating factor, hypoxemic respiratory failure, methicillin-resistant staphylococcus aureus, secondary pulmonary infection, transbronchial lung biopsy, whole-lung lavage

## Abstract

Pulmonary alveolar proteinosis (PAP) is a rare pulmonary disorder characterized by abnormal surfactant accumulation due to defective alveolar macrophage clearance. It often presents with exertional dyspnea and nonspecific imaging findings, commonly leading to diagnostic delays. We present the case of a 56-year-old woman with progressive hypoxia initially misattributed to acute respiratory distress syndrome, noncardiogenic pulmonary edema, and hypersensitivity pneumonitis. Corticosteroids were initiated based on early differential considerations. Further evaluation revealed concurrent methicillin-resistant *Staphylococcus aureus* (MRSA) pneumonia and bronchoalveolar lavage findings suggestive of PAP. Following stabilization and treatment of the infection, transbronchial biopsy confirmed PAP. The patient required prolonged intensive care and was ultimately transferred for whole-lung lavage. This case highlights the critical importance of early recognition of PAP in patients with characteristic imaging findings and progressive respiratory failure. Misdiagnosis can lead to inappropriate therapies and increased risk of infectious complications.

## Introduction

Pulmonary alveolar proteinosis (PAP) is a pulmonary disease characterized by alveolar surfactant accumulation due to defective clearance by alveolar macrophages [1]. It is rare, with a prevalence of around seven cases per million people [2]. Among affected individuals, there is a high prevalence of smokers and exposure to inhaled dust [3,4]. Three primary pathways lead to PAP: autoimmune (90% of cases), secondary, and hereditary. The pathophysiology is linked to granulocyte macrophage colony-stimulating factor (GM-CSF), a cytokine essential for alveolar macrophage function [5,6].

Clinical symptoms are often nonspecific, and 31% of patients are asymptomatic. When symptomatic, exertional dyspnea and cough are the most common manifestations, while fever and weight loss are rare [4]. Chest X-ray typically reveals bilateral opacities with consolidation or air bronchograms in a perihilar and basal distribution. On high-resolution computed tomography (CT) of the chest, a characteristic but nonspecific “crazy paving” pattern is observed, consisting of intra-alveolar ground-glass opacities and interlobular septal thickening [3,7]. Large focal airspace disease, mediastinal adenopathy, and pulmonary nodules are rare and should raise suspicion for underlying malignancy or infection [7].

Bronchoalveolar lavage (BAL) is diagnostic, and most cases can be diagnosed without lung biopsy [1,3]. The lavage fluid is typically milky, and histologic findings include large, foamy alveolar macrophages, eosinophilic bodies and granules, and proteinaceous material with positive periodic acid-Schiff (PAS) staining and negative Alcian blue staining [1]. Autoimmune PAP can be confirmed through the detection of anti-GM-CSF antibodies via enzyme-linked immunosorbent assay [1].

Whole-lung lavage remains the standard treatment, resulting in clinical and radiographic improvement in most cases [1]. For autoimmune PAP, subcutaneous and inhaled GM-CSF therapies offer less invasive treatment approaches that have demonstrated therapeutic efficacy [8-11].

## Case presentation

**Figure 1:**
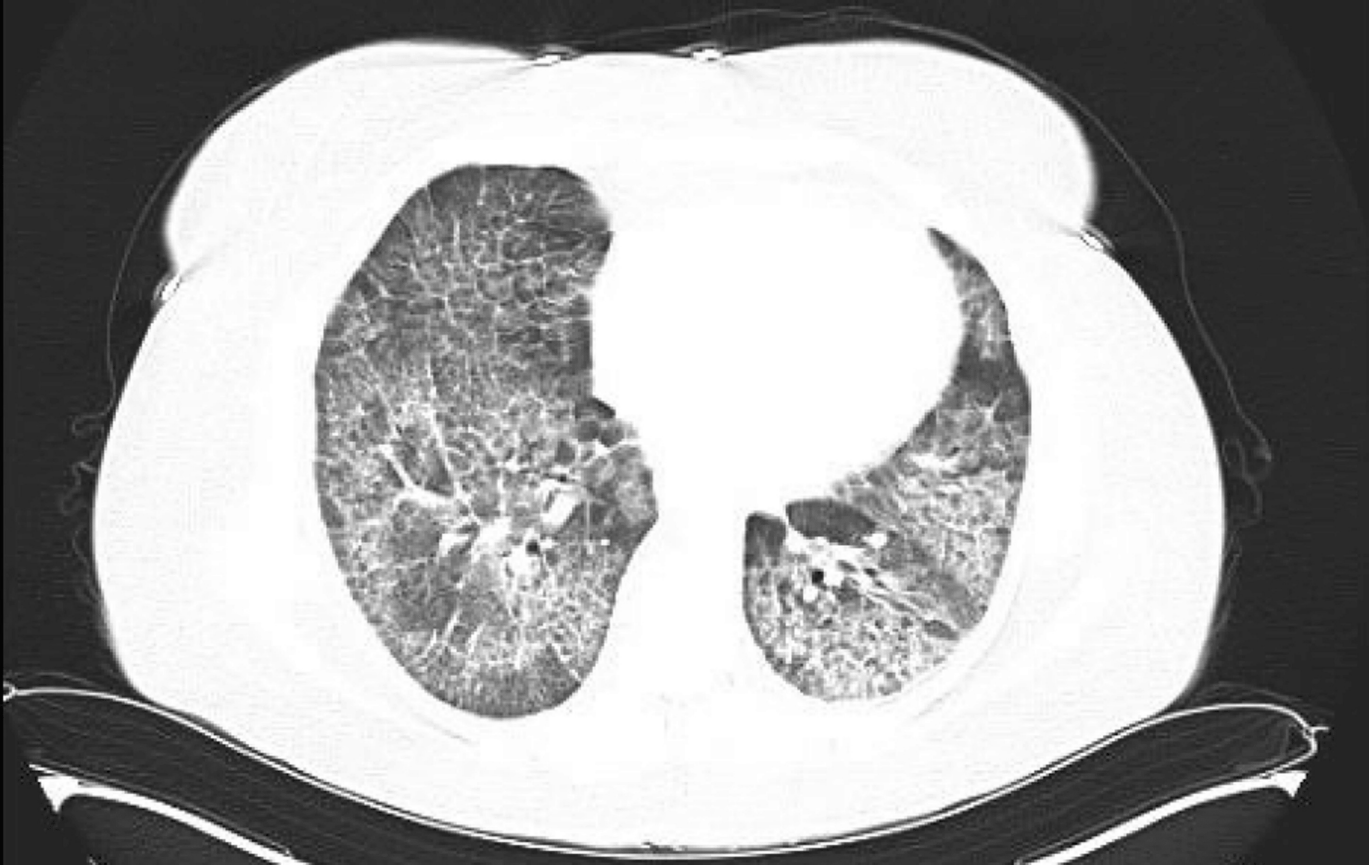
Computed tomography (CT) of chest showing a characteristic “crazy paving” appearance.

A 56-year-old woman with a medical history of non-insulin-dependent diabetes mellitus, hypertension, and prior *Nocardia* infection presented to the pulmonology clinic with progressive shortness of breath, first noted two months before admission. She was a lifetime non-smoker. She reported dyspnea on exertion and significant hypoxia during ambulation but denied cough or fever. A chest radiograph by her primary care physician showed abnormalities, leading to a referral to a pulmonologist. A high-resolution chest CT revealed severe diffuse lung disease with ground-glass opacities with a “crazy paving” appearance, though subpleural disease was absent (Figure 1). Differential diagnoses included recurring *Nocardia* infection, acute respiratory distress syndrome (ARDS), noncardiogenic pulmonary edema, and hypersensitivity pneumonitis.

The patient was admitted for further evaluation. On admission, the physical exam revealed poor bilateral air entry with inspiratory crackles; other findings were unremarkable. Laboratory tests showed elevated hemoglobin (17.7 g/dL, normal 12-16 g/dL) and elevated hematocrit (53.7%, normal 36-48%), consistent with polycythemia likely secondary to chronic hypoxia. Arterial blood gas from the clinic showed hypoxemic respiratory failure with a pH of 7.365, partial pressure of oxygen (pO₂) of 50 mmHg, and partial pressure of carbon dioxide (pCO₂) of 29.8 mmHg.

**Figure 2:**
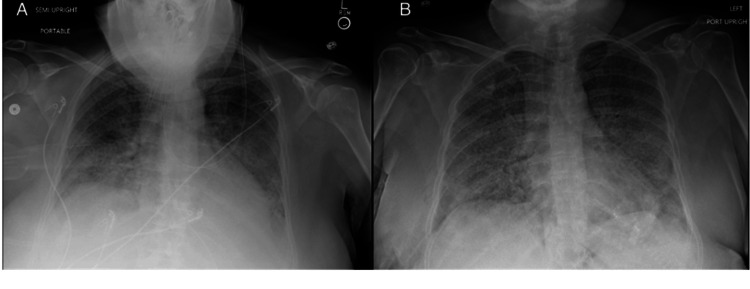
Chest X-rays showing severe diffuse lung infiltrates on hospital day 2 (A) and day 21 (B).

A fiberoptic bronchoscopy with BAL was performed, with white, opaque lavage fluid samples obtained from the right middle lobe and left upper lobe. Due to severe hypoxia, transbronchial biopsies were deferred. Following the procedure, the patient could not be extubated and required transfer to the intensive care unit (ICU) for mechanical ventilation. On day two of hospitalization, the patient was extubated and placed on a high-flow nasal cannula to manage oxygenation. Chest X-rays showed persistent diffuse bilateral infiltrates with no pneumothorax or large pleural effusions (Figure 2). The patient's imaging findings raised suspicion for PAP, given the radiological findings and oxygenation challenges. The patient also tested positive for methicillin-resistant *Staphylococcus aureus* (MRSA) pneumonia with respiratory cultures obtained at the time of bronchoscopy. She was started on a 10-day course of linezolid for MRSA coverage. She was also started on trimethoprim-sulfamethoxazole due to concern for recurring *Nocardia* infection. Despite treatment, the patient experienced fluctuating oxygen needs, requiring intermittent non-invasive ventilation.

**Figure 3:**
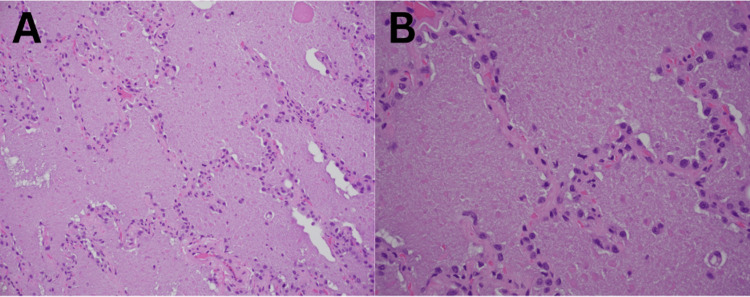
Micrograph showing alveoli filled with amorphous, eosinophilic debris containing occasional globules; hematoxylin & eosin (H&E) stain: medium power (A), high power (B).

**Figure 4:**
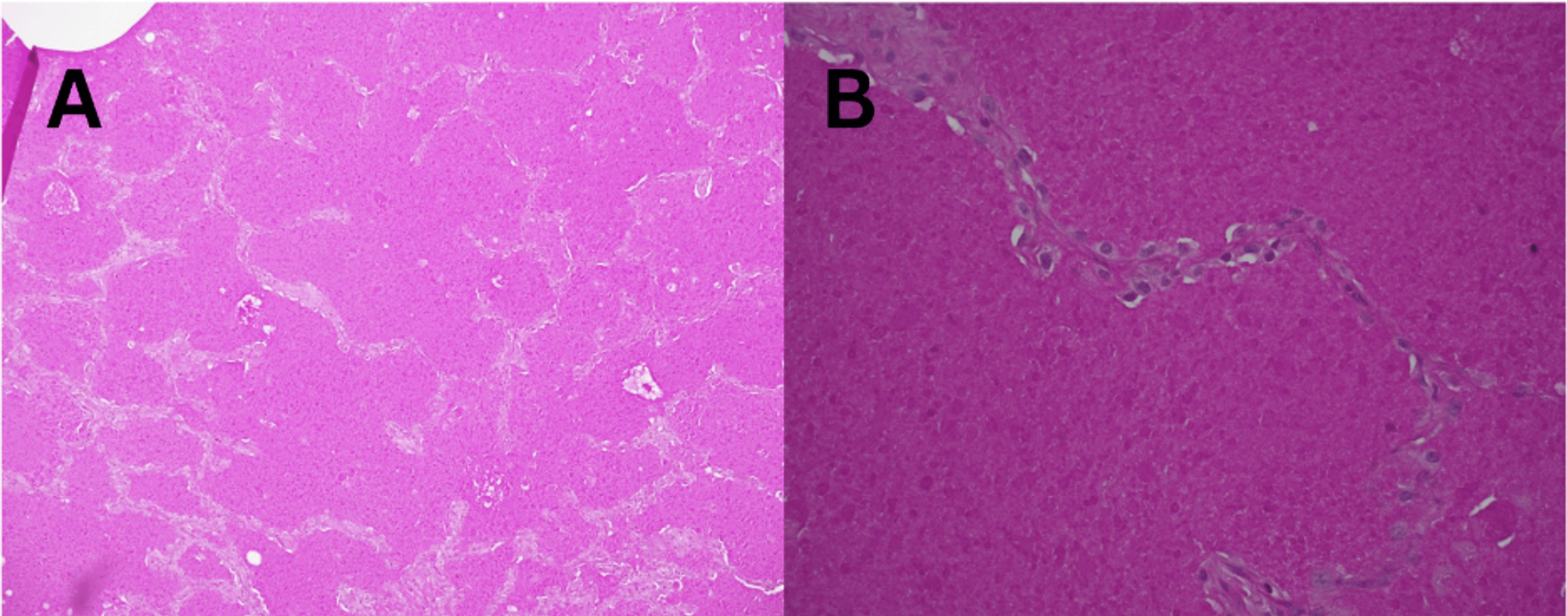
Positive periodic acid-Schiff staining of lung tissue; medium power (A), high power (B).

Close ICU monitoring and supportive care continued. Thoracic surgery was consulted regarding a lung biopsy, but conservative management with steroids was favored at first due to her instability. Over the following days, the patient's condition improved, and she completed treatment for MRSA pneumonia. At that time, transbronchial biopsies were completed and sent to pathology. Biopsy results demonstrated PAS-positive amorphous, eosinophilic material filling alveolar spaces, confirming the diagnosis of PAP (Figures 3-4). No organisms were seen on silver staining, ruling out nocardiosis. After stabilization, the patient was discharged from the ICU on day 27 of hospitalization. She was transferred to another facility for whole-lung lavage because the necessary equipment was unavailable at this facility.

## Discussion

PAP is a rare condition marked by alveolar surfactant accumulation in alveoli [1]. A large American study found the prevalence of PAP to be around seven cases per million people, with male and female individuals being affected equally. The prevalence is likely underestimated due to challenges and delays in diagnosis. PAP incidence increases with age, demonstrating a bimodal distribution peaking between ages 45-54 years and again at 65 years and older [2]. Patients with PAP also exhibit a higher burden of comorbidities, greater healthcare utilization, and significantly higher annual healthcare costs compared to matched controls [2].

Exertional dyspnea and cough are the most common symptoms, although approximately one-third of patients are asymptomatic [4]. Typically, PAP follows a progressive and indolent course. This case was distinctive due to the patient's severe hypoxemia, secondary polycythemia, and requirement for mechanical ventilation, an uncommon severity. The exacerbation of symptoms was likely due to a combination of underlying PAP and secondary MRSA pneumonia.

Diagnosing PAP presents significant challenges due to its nonspecific clinical features and overlapping imaging findings. The patient’s initial differential diagnosis included ARDS, noncardiogenic pulmonary edema, and hypersensitivity pneumonitis. These conditions can exhibit similar imaging features, such as ground-glass opacities and interlobular septal thickening, commonly referred to as the "crazy paving" pattern [12]. In PAP, this pattern arises from the accumulation of surfactant and PAS-positive proteinaceous material in the alveoli, leading to impaired gas exchange and respiratory symptoms [13]. The broad differential and diagnostic uncertainty in the current case led to the initiation of corticosteroid therapy, which can worsen autoimmune PAP severity and increase the risk of infections [14].

The patient's prior history of nocardiosis further complicated the diagnostic evaluation. Clinically, nocardiosis typically presents with a cough that may start dry but progresses to purulent sputum or hemoptysis [15]. In PAP, the cough is often initially non-productive but can worsen as surfactant accumulation increases [16]. On imaging, both conditions can show bilateral reticulonodular opacities [12,17], though cavitary lesions are more suggestive of nocardiosis, particularly in immunocompromised individuals [17].

Recent advancements in PAP treatment have introduced novel therapeutic options [8-11]. Inhaled GM-CSF therapy (molgramostim) demonstrated significant improvements in alveolar-arterial oxygen gradients and functional status in patients with autoimmune PAP in a multicenter randomized controlled trial [18]. A subsequent phase three clinical trial confirmed molgramostim’s ability to improve lung function and its favorable tolerability profile [19]. Additionally, a plasmapheresis protocol with a measurable decrease of anti-GM-CSF antibodies demonstrated reduced symptoms and improved lung function, resulting in the reduction of whole-lung lavage frequency [20].

Longitudinal data from a Japanese cohort highlight the variability in PAP’s natural history [4]. Among those diagnosed within one year, 66% experienced unchanged symptoms. In patients with intermediate disease duration (1-10 years), 42.5% improved, 29.8% worsened, and 27.7% remained unchanged. In individuals with disease exceeding 10 years, nearly two-thirds experienced worsening symptoms. Notably, 28.2% of asymptomatic individuals experienced spontaneous improvement without requiring whole-lung lavage [4].

This case underscores the importance of maintaining a broad differential diagnosis in patients presenting with progressive hypoxemia and characteristic “crazy paving” patterns. Early recognition and management of PAP are critical to optimizing patient outcomes and preventing unnecessary therapeutic interventions.

## Conclusions

In this report, we presented a rare, severe manifestation of PAP in a 56-year-old woman, complicated by secondary MRSA pneumonia. Her symptoms of severe hypoxemia, secondary polycythemia, and eventual requirement for mechanical ventilation underscore a rare potential of the disease to escalate in complexity. Challenges in diagnosis led to the initiation of corticosteroid therapy, which can worsen autoimmune PAP disease severity and increase infection risk. This highlights the importance of timely and accurate diagnosis in PAP patients. Inhaled GM-CSF shows promise in providing a less invasive option for autoimmune PAP management and offers a potential treatment alternative in locations where equipment for whole-lung lavage is lacking.
